# Partners in light: Vitamin E and the price of being green

**DOI:** 10.1093/plphys/kiaf251

**Published:** 2025-06-13

**Authors:** Thomas Depaepe

**Affiliations:** Assistant Features Editor, Plant Physiology, American Society of Plant Biologists; Laboratory of Functional Plant Biology, Department of Biology, Faculty of Sciences, Ghent University, Gent B-9000, Belgium

It's not easy being green. Plants are photosynthetic organisms that have the remarkable capacity to produce their own food using just sunlight and carbon dioxide. To achieve this, they rely on the efficient transfer of energy from photons to chlorophyll molecules, followed by electron transport reactions across the thylakoid membrane of chloroplasts. This process generates a proton gradient that drives ATP synthesis. However, under non-optimal growth conditions such as excess light, this system can become overloaded. Electrons may escape the electron transport chain and react with oxygen, producing reactive oxygen species (ROS; Foyer and Hanke 2022). While ROS can serve signalling functions, high levels are harmful and can damage proteins, DNA, and lipids. Fortunately, plants have evolved a suite of defence mechanisms to neutralize ROS, including antioxidants and other photo-protective systems. One particularly dangerous form of ROS is singlet oxygen, which, unlike other ROS, are not formed by simple electron transfer (Krieger-Liszkay and Trebst 2006). Instead, singlet oxygen is an excited state of molecular oxygen (triplet ground state) and is typically generated by energy transfer from excited chlorophyll molecules. Due to its high reactivity, it is especially damaging, notably through its ability to initiate lipid peroxidation. Moreover, conventional ROS quenching mechanisms are generally ineffective against singlet oxygen. The only known antioxidants capable of directly quenching singlet oxygen in chloroplasts are tocochromanols, a group of lipid-soluble antioxidants commonly known as vitamin E (Ferretti et al. 2018). This makes vitamin E essential for preventing photo-oxidative damage and ensuring plant survival.

Tocochromanols are produced exclusively by photosynthetic organisms, from cyanobacteria and algae to flowering plants, with different forms existing, such as tocopherols and tocotrienols (Esteban et al. 2009). It has long been assumed that their primary role is to protect the photosynthetic machinery. However, whether tocochromanols are strictly essential for survival in photosynthetic organisms has been debated (Mesa and Munné-Bosch 2023), especially since plants possess alternative photo-protective mechanisms, such as the xanthophyll cycle. In addition, loss of tocochromanols does not always lead to severe defects in growth or survival. So why have most photosynthetic organisms retained the capacity to make vitamin E? An answer might be found throughout the assessment of plant evolutionary history. In a recent paper published in *Plant Physiology*, Jené and Munné-Bosch (2025) investigated the function of vitamin E by studying parasitic plants. These species have independently evolved multiple times within flowering plants and include hemiparasites (which retain some photosynthetic activity) and holoparasites (which are entirely dependent on their hosts). The gradual loss of photosynthetic capacity has reduced selective pressure on photosynthesis-related genes. Therefore, Jené and Munné-Bosch hypothesized that this natural gradient in photosynthetic capacity could reveal the essential roles of vitamin E.

To test this, the authors started by analyzing chlorophyll and vitamin E content, along with markers of lipid peroxidation, in a range of parasitic plants. As expected, species with lower photosynthetic capacity had reduced chlorophyll levels, which was paralleled by a decrease in vitamin E levels. Hemiparasites as well as holoparasites, which no longer actively perform photosynthesis, also showed reduced lipid peroxidation, likely because of their diminished risk of photo-oxidative stress. These findings suggest an evolutionary association where loss of photosynthetic capacity is linked to reduced vitamin E content (Figure). Yet, both hemiparasites and some holoparasites, including field dodder (*Cuscuta campestris*), still retain considerable levels of α-tocopherol, the major type of vitamin E. To further explore vitamin E functions in field dodder, the authors examined its interaction with the common host lentil. After several weeks of infection, lentil plants showed increased sensitivity to light stress: elevated nonphotochemical quenching, increased lipid peroxidation, and decreased chlorophyll content, all signs of oxidative damage. These symptoms were accompanied by a rise in vitamin E levels, likely in response to the increased oxidative stress. On the other hand, while field dodder no longer actively photosynthesizes, it did display some residual light-dependent electron transport, particularly at low light intensities. This modest activity still triggered photo-protective responses, including increased vitamin E production. This suggests that even minimal photosynthetic activity in field dodder generates enough ROS to require antioxidant protection. These mechanisms were especially critical in its most vulnerable stages: the initial free-living phase and during the formation of haustoria (structures that connect to the host). The authors confirmed vitamin E's protective role in these stages using an oxidative stress assay on isolated chloroplasts from field dodder, showing a clear relationship between oxidative damage and vitamin E levels.

**Figure.**
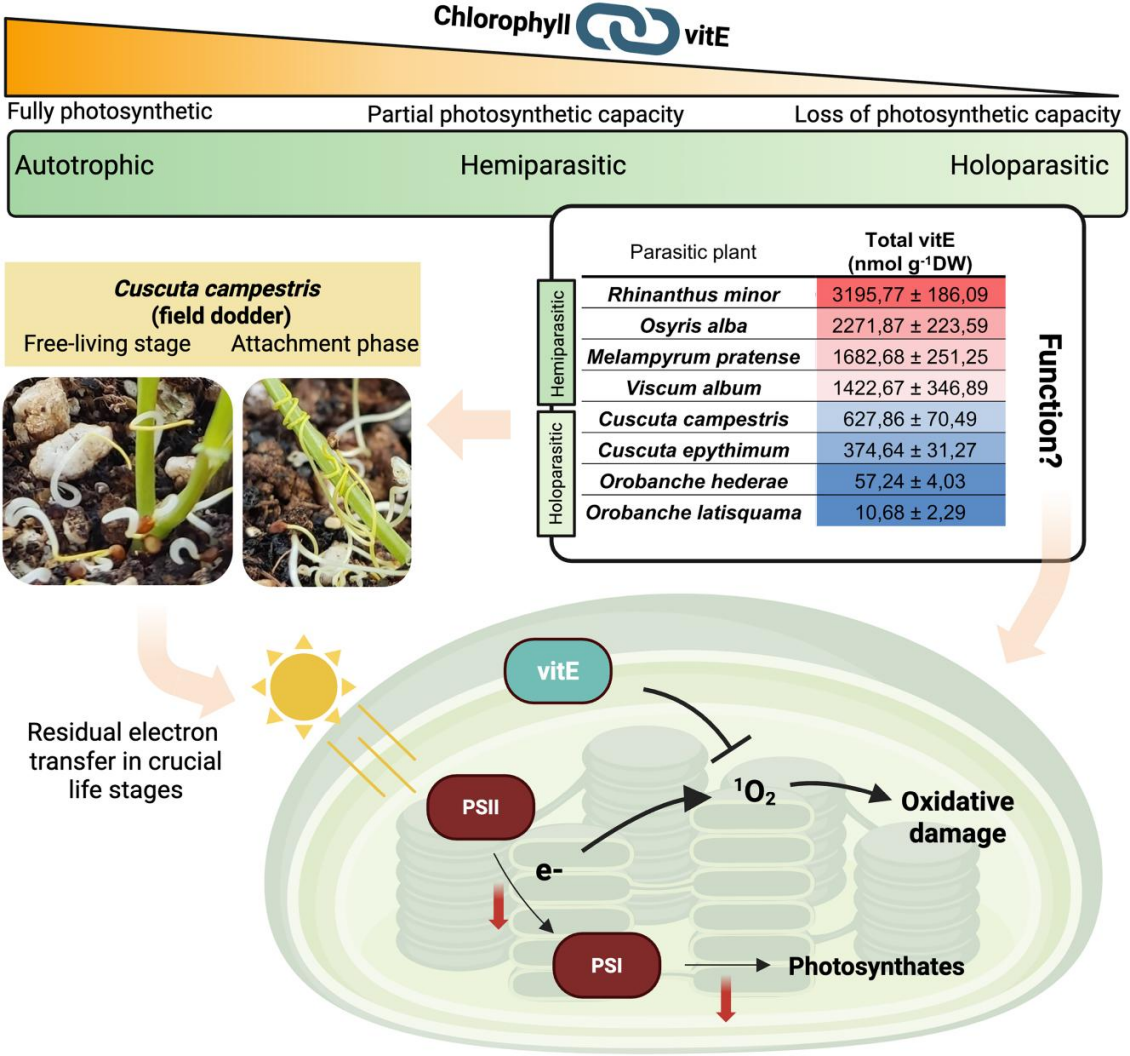
Evolutionary association between photosynthetic capacity and vitamin E. A reduction in photosynthetic capacity is paralleled by reductions in the antioxidant vitamin E (vitE). Parasitic plant species, like field dodder (*Cuscuta campestris*), retain certain amounts of vitE, the function of which is thought to play a role in its sensitive but crucial free-living stage and during attachment with its host. In chloroplasts, a low rate of photosynthesis and associated electron transport, indicated by red downward arrows, is sufficient to produce singlet oxygen (^1^O_2_), especially under low light conditions. Chloroplast-derived vitE limits oxidative damage by quenching ^1^O_2_. Figure was partially adapted from Jené and Munné-Bosch (2025) and was prepared in Biorender.

In conclusion, Jené and Munné-Bosch have tackled one of the many outstanding questions surrounding vitamin E's function in photosynthetic organisms. Their inventive approach, drawing on the natural diversity in photosynthetic capacity among parasitic plants, elegantly demonstrated the evolutionary persistence and functional necessity of vitamin E. Their findings reinforce the idea that vitamin E is crucial not only in fully photosynthetic organisms but also in those on the fringe, protecting them in transitional, high-risk stages where other antioxidant systems may be insufficient. In particular, the retention of vitamin E biosynthesis in parasitic plants like field dodder underscores its fundamental role in managing photo-oxidative stress, even when photosynthesis is minimal. This study highlights an evolutionary association between vitamin E, photosynthesis, and photoprotection—a triad that has helped plants survive in high-light, oxygen-rich environments. Future work could investigate whether field dodder retains other photo-protective systems and what other functions vitamin E might have, drawing on the available genomic and transcriptomic resources (Vogel et al. 2018; Bawin et al. 2022). While we still have much to learn about this powerful antioxidant, one thing is clear: although being green comes with risks, all plants can count on vitamin E to help them survive the light.

## Data Availability

No new data were generated or analyzed in support of this research.
